# Electronic protocol for clinical data collection in chronic visceral ischemia

**DOI:** 10.1590/1677-5449.006916

**Published:** 2017

**Authors:** Adriana Buechner de Freitas Brandão, Jorge Rufino Ribas Timi, Osvaldo Malafaia

**Affiliations:** 1 Instituto da Circulação, Curitiba, PR, Brazil.; 2 Faculdades Pequeno Príncipe – FPP, Department of Surgery, Curitiba, PR, Brazil.; 3 Universidade Federal do Paraná́ – UFPR, Department of Surgery, Curitiba, PR, Brazil.; 4 Faculdade Evangélica do Paraná́ – FEPAR, Department of Surgery, Curitiba, PR, Brazil.

**Keywords:** database, data collection, mesenteric ischemia, renovascular hypertension, base de dados, coleta de dados, isquemia mesentérica, hipertensão renovascular

## Abstract

Patients with vascular diseases present with a long medical history which makes for complex and confusing medical records. Electronic record systems have a large storage capacity and high information processing capabilities, and may therefore improve the quality and reliability of prospective clinical studies. In the present study, a theoretical framework for clinical data collection in chronic visceral ischemia was created containing 5706 items, organized into six major categories: medical history, physical examination, laboratory testing, diagnosis, treatment and outcome. The database was used to construct an electronic data collection protocol, which was uploaded into the Integrated Electronic Protocol System (Sistema Integrado de Protocolos Eletrônicos, SINPE©) and then used to perform retrospective collection and analysis of clinical data from 10 patients using the SINPE© analysis module.

## Resumo

Pacientes portadores de doenças vasculares possuem histórico médico longo, o que dificulta a revisão das informações contidas nos prontuários. Protocolos eletrônicos de dados clínicos permitem grande capacidade de armazenamento e processamento de informações, otimizando a qualidade e a confiabilidade de trabalhos científicos. Neste trabalho foi criada uma base teórica para coleta de dados clínicos referentes à isquemia crônica visceral composta de 5.706 itens agrupadas sob os seguintes categorias principais: anamnese, exame físico, exames complementares, diagnóstico, tratamento e evolução. Essa base foi transformada em um protocolo eletrônico de coleta de dados, e este foi incorporado ao Sistema Integrado de Protocolos Eletrônicos (SINPE©) e utilizado para cadastrar, coletar e analisar os dados de dez pacientes através do módulo SINPE© Analisador.

## INTRODUCTION

Patients with vascular disease often have a long medical history of symptoms, comorbidities, risk factors, and hospitalizations. This may hinder the extraction of patient data from medical records. Electronic medical record systems have a large storage capacity, high information processing capabilities, and facilitate the access to and retrieval of patient information. Such systems may therefore lead to significant improvements in the quality and reliability of prospective clinical studies.

The first attempts to integrate computers and information technology into biology and medicine were made in the 1950s. It was then that two pioneers in the area of health information systems, Robert S. Ledley and Lee B. Lusted, wrote seminal articles in the influential American journal Science, in which they encouraged researchers in biology and medicine to adopt information technology.^1^ These articles marked the beginning of a series of discussions on the use of computers in medicine.^2^ In the 1960s, computer use was restricted to administrative, operational and financial purposes.^3^^,^ 4 However, from that point onwards, information technology gradually began to be used for the management of clinical, radiology and hemodynamic laboratories,^5^ drug prescriptions, and diagnostic registration.^6^

In the past two decades, significant efforts have been made to expand the use of information technology in medicine, and special attention has been paid to electronic substitutes for paper records, which could facilitate the archiving and retrieval of information for clinical research, increasing its comprehensiveness and reliability.^7^^,^ 8 The use of electronic records leads to major improvements in the quality of scientific research by facilitating data collection, organization, storage, and retrieval.^9^

Atherosclerosis is the most common cause of chronic visceral ischemia, and the most frequent pathology in individuals aged 60 years or older. However, chronic visceral ischemia syndrome is rare in the general population. Even large-scale centers have difficulty locating a sufficient number of cases for clinical research.^10^

Although infrequent, this condition has severe consequences for those affected, which underscores the need to optimize the collection of data from patients with the condition. The aims of the present study were as follows:

a) To construct a theoretical framework for clinical data collection in chronic visceral ischemia;

b) To reproduce this information electronically in the form of a computer program;

c) To incorporate this program into the Integrated Electronic Protocol System (Sistema Informatizado de Protocolos Eletronicos, SINPE©);

d) To evaluate the protocol and the SINPE© analysis module through a pilot study.

## MATERIAL AND METHODS

This study was approved by the institutional Research Ethics Committee under registration number 327.EXT004/2010-01, and written informed consent was obtained from all participants.

The Electronic Protocol for Clinical Data Collection in Chronic Visceral Ischemia was developed through a descriptive study, which, for explanatory purposes, can be divided into six stages:

a) Construction of a theoretical framework for clinical data collection in chronic visceral ischemia;

b) Development of a data collection protocol using text editing software;

c) Formatting protocol data for the construction of an electronic database;

d) Incorporation of the electronic protocol into the SINPE©;

d) Evaluation of the protocol through a pilot study;

f) Pilot study data analysis.

After the definition of our main object of study (Chronic Visceral Ischemia), we sought to identify and collect all relevant data from standard textbooks in the area, such as Peripheral Vascular Disease,^11^ Vascular Surgery,^10^ and Vascular Surgery.^12^ Electronic databases such as MEDLINE, LILACS and SCIELO, were also searched for relevant articles and dissertations published in the past 5 years. These data were used to develop a main protocol divided into two sections: chronic intestinal ischemia and renovascular hypertension. These instruments would then be formatted for electronic submission.

Each of the two sections in the protocol was organized in six hierarchical categories: patient history, physical examination, laboratory examinations, diagnosis, treatment and evolution. Each of these was, in turn, composed of a series of sub-items. The items were then transposed to an electronic medium using Microsoft Word and Microsoft Excel, and arranged into the standard format required by SINPE©.

The management of electronic protocols was performed using the SINPE© platform, a Windows-based tool constructed using a .NET Framework©, the C#(C-Sharp) programming language, and databases hosted on Microsoft Access© or the Microsoft SQL Server©.

After data conversion and the installation of all required software, the data collection protocol was incorporated into SINPE© with the help of the MIGRASINPE© package.

With this tool, we were able to create a data collection protocol and a Microsoft Access database using the previously mentioned text file containing all hierarchical items developed based on our literature review.

The pilot study of the electronic protocol was performed in a major hospital in southern Brazil. Data were collected retrospectively from a sample of 10 patients treated for chronic visceral ischemia, with the aim of evaluating the applicability of our protocol, and validating it for use in data collection for scientific research.

The information collected in the pilot study was analyzed using the SINPE Analyzer© package. This tool provides an interface for the rapid visualization of electronic protocol data in SINPE©, and for automatic and instantaneous figure plotting, statistical testing, printing, saving and exporting results.

Protocol functionality and system performance were evaluated throughout the study.

## RESULTS

The literature review gave rise to 5706 items, which were organized into six major hierarchical categories, which are shown in Table 1. These categories served as the basis for an item tree structure, whose first level contained all six major items, while subsequent levels comprised the sub-items included in each scale. Each major category included approximately six or seven sub-items. A sample of the item structure used in the protocol is shown in Table 2. In extreme cases, a major category could include up to 13 sub-items.

**Table 1:** Main items in the data collection framework.

**Categories**	**Sub-items (children)**
Medical history	962
Physical examination	1,271
Laboratory examinations	2,756
Diagnosis	18
Treatment	525
Evolution	167

**Table 2:** Sample of the data structure within the theoretical framework.

**Level**	**Main Item**	**Sub-item**	**Sub-item**	**Sub-item**	**Sub-item**
	MEDICAL HISTORY				
2		SYMPTOMATIC			
3			PAIN		
4				ABDOMINAL	
5					POSTPRANDIAL

**Figure 1:**
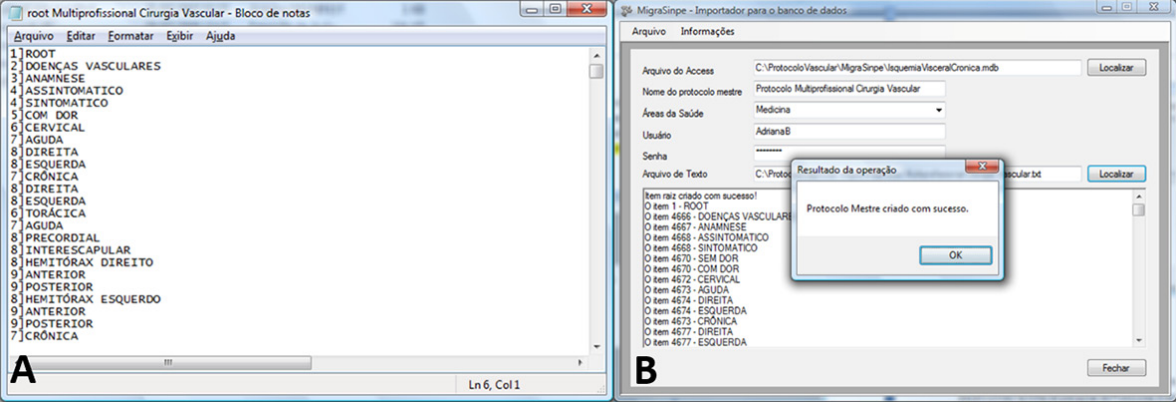
(A) Text file containing the computerized version of the main protocol. The data structure of the protocol can be observed through the outlined items and their associated hierarchical structure number. This list will be used as input data for the creation of the protocol; (B) MIGRASINPE® parameter selection screen, and popup window confirming the successful creation of the main protocol, after the data import from the original text file.

The theoretical framework was converted into an electronic format using Microsoft Word and Microsoft Excel. The electronic protocol created using Microsoft Word was then converted into a text file (Figure 1A), and incorporated into SINPE© using the MEGASINPE© package.

Once all relevant parameters had been selected, the data began to be imported into the system. At the end of the process, a popup window titled “Operation results” confirmed that the main protocol had been successfully created (Figure 1B).

**Figure 2:**
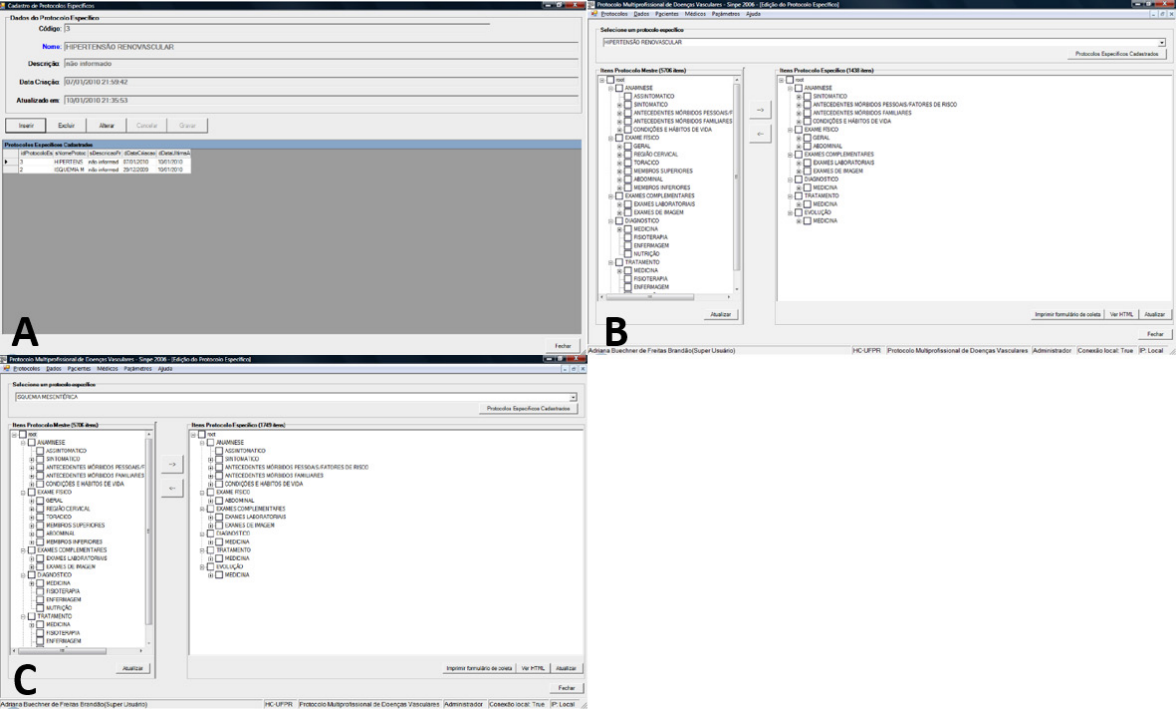
(A) Specific protocol registration screen on the SINPE© platform. From the original main protocol, two specific protocols were created: Mesenteric Ischemia protocol and Renovascular Hypertension protocol; (B) Specific protocol editing screen displaying all items in the Mesenteric Ischemia protocol; (C) Specific protocol editing screen displaying all items in the Renovascular Hypertension protocol.

Once this document was created by the MIGRASINPE© package, it could be accessed from within the SINPE©, allowing for the creation of the two additional protocols pertaining to renovascular hypertension and mesenteric ischemia (Figure 2A).

Specific protocols can be created using the Protocols/Specific command. A Name and Description for the new protocol must first be provided, before the main protocol items which are to be included in the new document are selected in the Specific Protocol Editing window. The renovascular hypertension and mesenteric ischemia protocols comprised 1438 and 1749 items, respectively, from the main protocol (Figure 2B and 2C).

**Figure 3:**
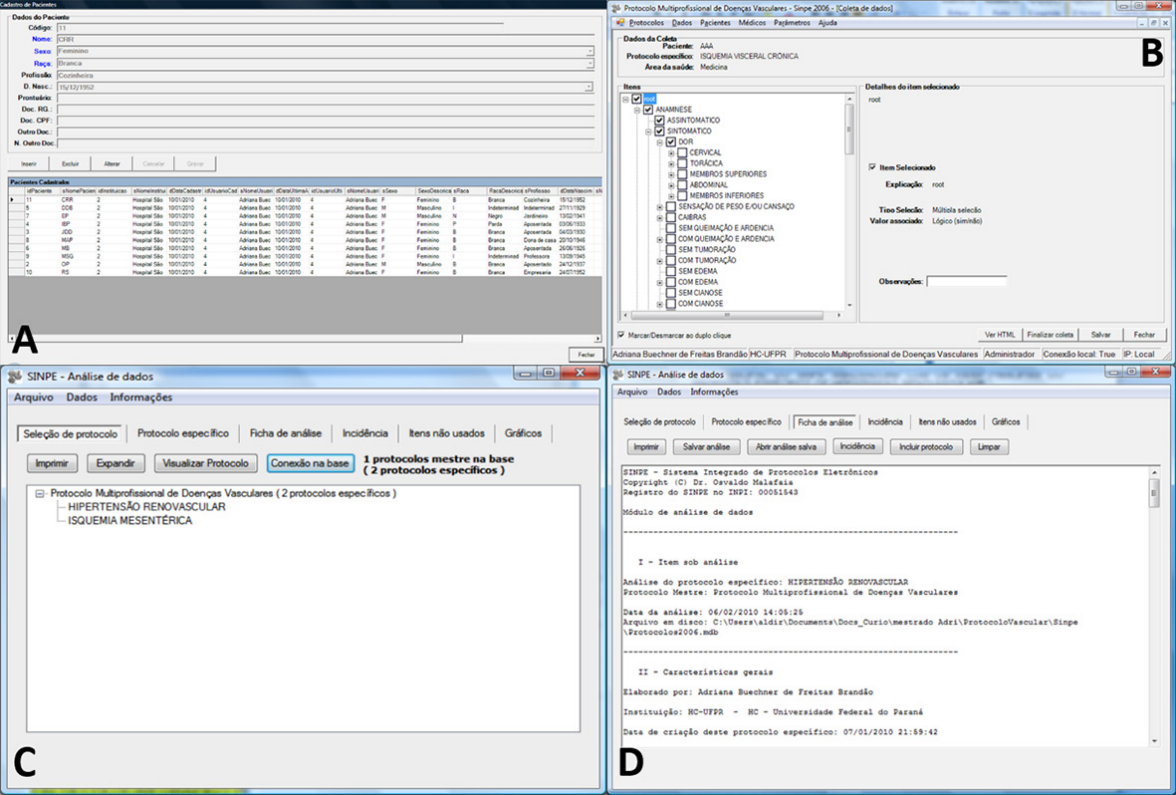
(A) Patient registration screen on the SINPE® software. At the top of the screen, patient data fields can be seen and edited, and at the bottom, a list of all the participants in the pilot study can observed; (B) SINPE® data collection screen. This is the screen used for data entry, all the observed items are checks in the form; (C) Main window of the SINPE Analyzer®, with the specific protocol for further analysis and report; (D) Main window of the SINPE Analyzer®, displaying the data in the analysis report. The file contains general information of the protocol.

The data collected in the pilot study was then uploaded to the system. The following data were collected for each patient: Name (abbreviated), Gender, Race, Occupation, and Birth date (Figure 3A). Data were collected from a total of 10 patients. The renovascular hypertension protocol was used in eight cases, while the mesenteric ischemia protocol was used in the remaining two (Figure 3B). The small size of our sample can be attributed to the rarity of the diseases investigated.

Data were analyzed using the SINPE Analyzer© package. All of the graphical, statistical, printing, storage and data exportation functions available in the software were used and evaluated (Figure 3C).

The main menu of the SINPE Analyzer© software displays three options: *File, Data and Information*.

Once a specific protocol is selected, a series of operations can be performed, such as:

1) View Protocol, which can display all main items and respective sub-items, print the protocol in text or graphical format, and expand/collapse all items.

2) View the Analysis Report, which displays all items under analysis (name of the specific protocol and associated main protocol, date of analysis and name of the file analyzed), provides general protocol characteristics (developer, institution, date of development, last revision, protocol sections and number of observations), and describes the data collection process (number of observations, beginning and end dates of data collection, number of collaborators, number of participating institutions and patients) (Figure 3D).

**Figure 4:**
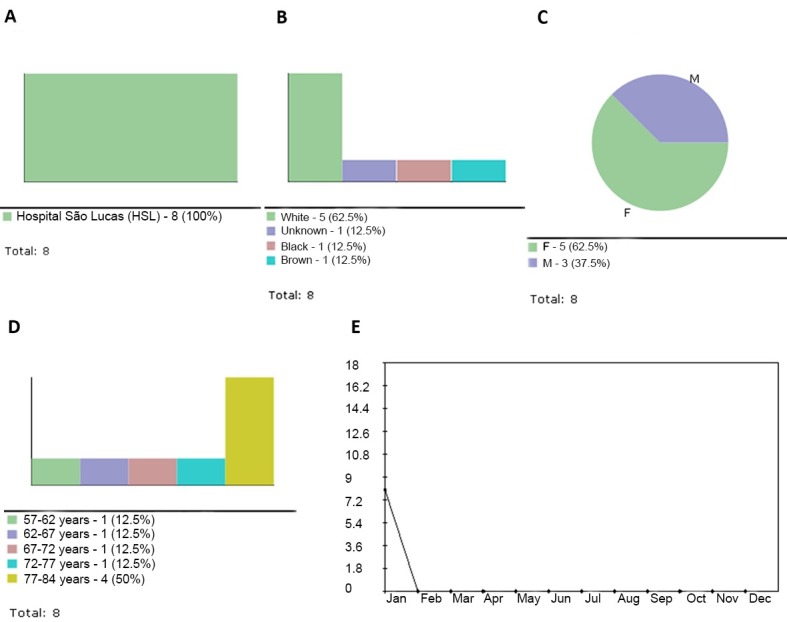
(A) Patient distribution by institution; (B) Patient distribution by race; (C) Patient distribution by gender; (D) Patient distribution by age range; (E) Patient distribution per time period.

**Figure 5:**
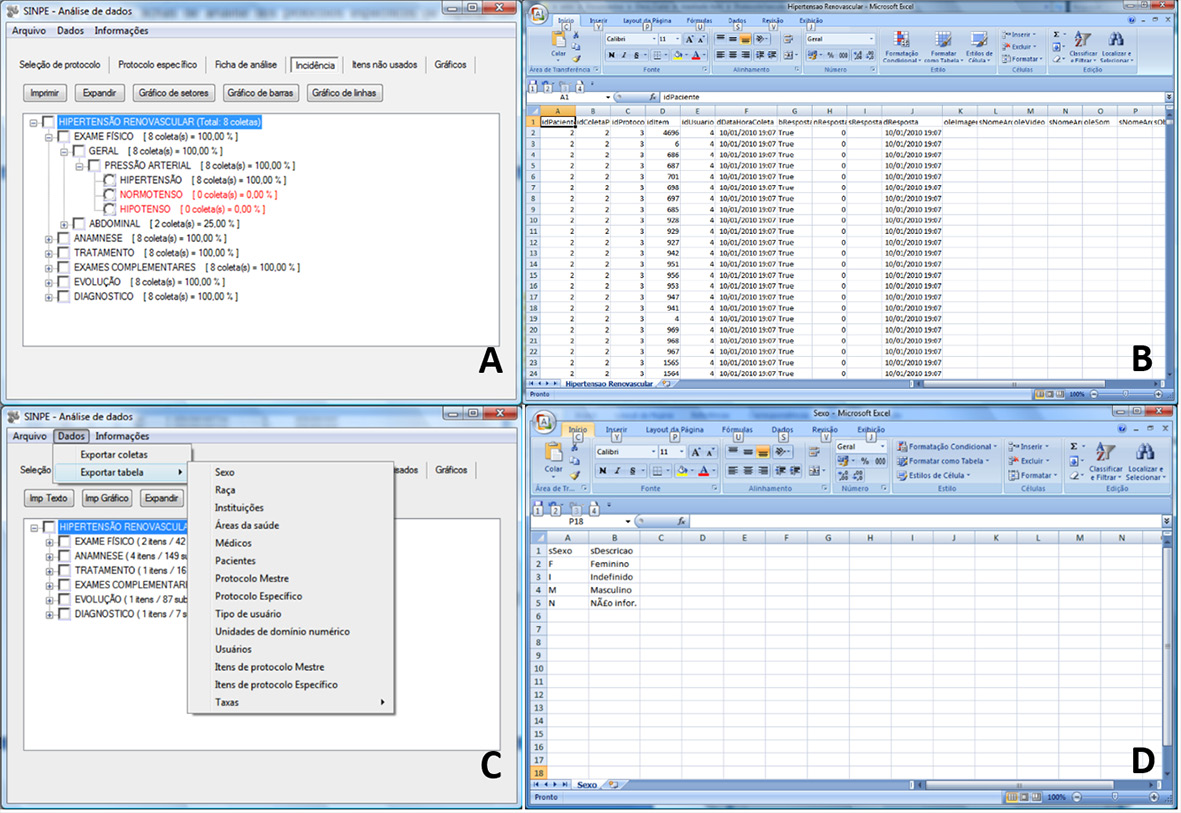
(A) Main window of the SINPE Analyzer®, displaying incidence data; (B) Sample CSV file containing all the data of Renovascular Hypertension protocol, exported by SINPE Analyzer®; (C) Dropdown menu showing SINPE Analyzer® export options; (D) Sample CSV file containing specific data (gender) of the Renovascular Hypertension protocol, exported by SINPE Analyzer®.

The Analysis Report option also allows for the generation of pie, bar or line graphs to illustrate the distribution of patient data per institution (Figure 4A), race (Figure 4B), gender (Figure 4C), age range (Figure 4D) and time period (Figure 4E). The figures shown below were created using data collected with the renovascular hypertension protocol. The Incidence sub-menu displays the frequency of each item in the sample, displayed as a percentage. Any items which are not observed in the sample are displayed in red (Figure 5A). The graphs, in turn, can be printed, saved, included in the analysis report or copied to the clipboard.

3) Export data. Data can be exported from the SINPE© in one of two ways. The first is the exportation of an entire dataset (Figure 5B), while the second pertains to the exportation of data from a single table (Figure 5C and 5D). Once the user selects the data to be exported, is selected, it must be given a file name with .csv extension.

Our assessment of data export functions in the SINPE Analyzer© revealed that this software package can facilitate the exchange of protocol information and patient data between different types of software. The information is displayed in an organized and reliable manner, in a standardized format which can be easily imported into other software packages.

## DISCUSSION

The standardization of data collection procedures is essential to ensure the quality of data analysis. Descriptive studies often face significant data limitations due to incomplete or inadequately documented patient records, which may compromise the quality of results.^5^^,^ 13

At first glance, electronic medical records may appear to be a simple way to ensure the absence of missing data, and the standardization of data collection, in retrospective clinical studies.^14^ However, even though the transition from paper to electronic records may lead to significant advantages, it must be performed with meticulous care. Coexisting paper and electronic records may differ in their contents by up to 7%.^7^^,^ 15

The literature review and the conversion of the item hierarchy from Microsoft Word to the SINPE© format were the most time-consuming and demanding phases of the study. The research team met several times during the construction of the file which would later become the main protocol for data collection in vascular surgery, and give rise to several specific protocols.

The greatest challenges faced in this study were the creation of a single summary file for all relevant information obtained in the literature review, and the development of questionnaire items which would adequately capture all concepts examined without repetition or redundancy. The protocol has only the relevant items for medical research in the area.

The development of a research protocol with a solid theoretical basis provides a structure to the research data and ensures that all information is collected in a coherent and standardized manner.

Recent years have seen a continued annual doubling of computer processing power, which translates into a decrease of approximately 50% in the costs of IT infrastructures. As a result, these have become increasingly and inevitable integrated into clinical research.^16^

As a general rule, the introduction of novel procedures which interfere with people's daily habits is not well accepted and slow to occur, requiring constant monitoring and training, as well as continuing education.

The implementation of electronic systems and protocols is riddled with challenges which one must be prepared to face. These include cultural resistance, poor strategic planning for system implementation, little organizational incentive, hospital autonomy, and, most importantly, poor healthcare planning at a population level.^16^ The development of an electronic protocol for clinical data collection in chronic visceral ischemia aimed to provide a computerized platform for the collection and analysis of clinical data, improving the quality of future research and contributing to the increasing integration of information technology and medicine.

The safety of patient data is also a major strong point of electronic records.

The SINPE© has several mechanisms which guarantee the confidentiality of patient data. These include mandatory user identification, user permission levels, and the impossibility of altering saved protocol data or editing research protocols after the end of data collection.

The MIGRASINPE© played an important role in the present study. Without it, all items in the main protocol would have had to be entered manually into the system.

These programs are easy to use, and facilitate the transfer of questionnaire items from the main item pool into specific protocols. Mistakes in the process can be easily corrected without losing any of the previously entered data.

The pilot study was performed in a hospital in Southern Brazil. Given the rarity of the diseases investigated, a retrospective study was performed. The aim of this procedure was to evaluate the applicability of the protocol and validate its use in the collection of clinical research data. Statistical significance testing was not among the goals of our study, since our sample was far too small for this purpose.

The data collection platform in the SINPE© system is similar to Microsoft Windows, and therefore familiar to most computer users. However, this does not eliminate the need for researcher training, especially since the program does not allow for the editing of saved data.

The SINPE© platform can also be accessed from client computers, Internet servers, and handheld devices, facilitating bedside data collection and remote access to research data. The protocols can also be printed on paper, allowing data collection to continue even in the presence of technical problems or power outages. Clinical databases might belong to a single institution or be shared by different research centers. Data collected within a single institution can be used to delineate the clinical profile of its target population, evaluate diagnostic and treatment procedures, and study the performance of health care professionals. When studying rare clinical conditions, it may be useful to ensure that research protocols are available in more than one institution.^17^

In the present study, we also evaluated the performance of the SINPE Analyzer© software. This program computes the distribution of patient data by institution, race, gender, age, and time period. This information can then be printed, stored or exported to another software program.

The software used in the present study generated statistical results quickly and efficiently, decreasing the financial and human resource burden of clinical research on health care institutions.

The SINPE© system has received favorable reviews from health care professionals, increased the productivity of scientific research, and decreased its time demands by 50%.

Recent years have seen a growing reliance on information technology in health care. These initiatives have received significant human, financial and organizational investments. However, there is still much to be done, and much to invest in terms of time and effort, before full integration is achieved. Nevertheless, as long as there is serious interest in improving the quality of clinical research, these investments are fully justified.

## CONCLUSIONS

The present study yielded several important results. A theoretical framework for clinical data collection in chronic visceral ischemia was successfully constructed, and reproduced in an electronic format in the form of a computerized protocol. This instrument was, in turn, uploaded to the SINPE© system, and evaluated through a pilot study in which data was collected and analyzed using the SINPE© Analyzer package.
